# Supplementary material for: Life span inequality as a function of the moments of the deaths distribution: Connections and insights
Source: PLoS One. 2022 Jan 31;17(1):e0262869. doi: 10.1371/journal.pone.0262869 (PMC8803175; doi:10.1371/journal.pone.0262869)
Supplement: S1 Appendix — A Mathematical Derivations [32]. B Supplementary Information [23]. (PDF) [file pone.0262869.s001.pdf]

A Mathematical Derivations

A.1 Life Table Functions

We now briefly review the relevant relationships between the life table functions μ, s, and f used in the Materials and Methods section. (We assume, as before, that these functions are at least differentiable.)

The survival function $s(x)$ is defined in terms of the mortality function $\mu(x)$ by

$$s(x) = e^{-\int_0^x \mu(t)\,dt}. \tag{26}$$

It follows that

$$s'(x) = -\mu(x)s(x).$$

One consequence of this is that the deaths distribution, $f(x) = \mu(x)s(x)$, can be alternatively expressed as $f(x) = -s'(x)$, yielding (2). Using this in (7) yields

$$e_0 = \int_0^\omega xf(x)\,dx = -\int_0^\omega xs'(x)\,dx = \int_0^\omega s(x)\,dx, \tag{27}$$

using integrating by parts. The last expression in (27) is precisely the definition of life expectancy at birth.

In terms of the central moments (8), for the first two ($n = 0$ and $n = 1$) we note that

$$m_0(a) = \int_a^\omega f_a(x)\,dx = \frac{\int_a^\omega f(x)\,dx}{\int_a^\omega f(x)\,dx} = 1, \tag{28}$$

and

$$\begin{aligned} m_1(a) &= \int_a^\omega (x - e_a)f_a(x)\,dx \\ &= \int_a^\omega xf_a(x)\,dx - e_a\int_a^\omega f_a(x)\,dx \\ &= e_a - e_a m_0 = e_a - e_a = 0. \end{aligned} \tag{29}$$

A.2 Proofs

Proof of Theorem 1. First, we choose a specific $a \in [0, \omega)$. We then construct from this the a-truncated deaths distribution $f_a(x)$ according to (3), and calculate the associated e_a value (from (7)). Next, we use the analyticity assumption on $e(x)$ to expand $e(x)$ in a Taylor series about $x = e_a$ (we stress, for clarity, that the a-value here is the same a-value used to construct the a-truncated deaths distribution f_a):

$$e(x) = e(e_a) + e'(e_a)(x - e_a) + \frac{e''(e_a)}{2!}(x - e_a)^2 + \frac{e'''(e_a)}{3!}(x - e_a)^3 + \cdots \tag{30}$$

$$= \sum_{n=0}^{\infty} \frac{e^{(n)}(e_a)}{n!}(x - e_a)^n. \tag{31}$$

(Since $0 \le e_a \le \omega$ and since $e(x)$ is analytic on $[0, \omega]$ (by assumption), we are guaranteed that the Taylor series on the right-hand side converges to $e(x)$ for every

$x \in [0, \omega]$.) Now, let us write (31) as

$$\begin{aligned} e(x) &= \sum_{n=0}^{k} \frac{e^{(n)}(e_a)}{n!}(x-e_a)^n + \sum_{n=k+1}^{\infty} \frac{e^{(n)}(e_a)}{n!}(x-e_a)^n \\ &=: \sum_{n=0}^{k} \frac{e^{(n)}(e_a)}{n!}(x-e_a)^n + R_k(x). \end{aligned} \tag{32}$$

By the analyticity of $e(x)$ we know that, for each $x \in [a, \omega]$, $R_k(x) \to 0$ as $k \to \infty$. Now, multiply (32) by $f_a(x)$ and integrate both sides over the interval $[a, \omega]$ to obtain:

$$\begin{aligned} e^{\dagger}(a) &= \int_a^{\omega} \left[\sum_{n=0}^{k} \frac{e^{(n)}(e_a)}{n!}(x-e_a)^n f_a(x) + R_k(x) f_a(x) \right] dx \\ &= \sum_{n=0}^{k} \frac{e^{(n)}(e_a)}{n!} \left[\int_a^{\omega} (x-e_a)^n f_a(x)\, dx \right] + \int_a^{\omega} R_k(x) f_a(x)\, dx \\ &= \sum_{n=0}^{k} \frac{e^{(n)}(e_a)}{n!} m_n(a) + \int_a^{\omega} R_k(x) f_a(x)\, dx \\ &= e(e_a) + \frac{e''(e_a)}{2!}\sigma^2(a) + \sum_{n=3}^{k} \frac{e^{(n)}(e_a)}{n!} m_n(a) + \int_a^{\omega} R_k(x) f_a(x)\, dx, \end{aligned} \tag{33}$$

where we used (8) in the penultimate equality and also made use of (28) and (29). We now show that the integral term in (33) tends to zero as $k \to \infty$.

To begin, we note that since f_a is assumed continuous on $[a, \omega]$ then it is bounded on that set, so that $|f_a(x)| \leq M$ for some $M > 0$ and for all $x \in [a, \omega]$. Thus,

$$\left| \int_a^{\omega} R_k(x) f_a(x)\, dx \right| \leq M \int_a^{\omega} |R_k(x)|\, dx \tag{34}$$

Next, since $e(x)$ is assumed analytic on $[0, \omega]$ then so is $R_k(x)$. In particular, this implies that $R_k(x)$ is continuous on $[0, \omega]$, as is $|R_k(x)|$, being a composition of continuous functions. Thus, the Mean Value Theorem for Integrals [32] guarantees the existence of a $c \in (a, \omega)$ such that

$$\int_a^{\omega} |R_k(x)|\, dx = |R_k(c)| \int_a^{\omega} 1\, dx = (\omega - a)|R_k(c)|.$$

Substituting this into (34) yields

$$\left| \int_a^{\omega} R_k(x) f_a(x)\, dx \right| \leq M(\omega - a)|R_k(c)|. \tag{35}$$

And because $R_k(x) \to 0$ as $k \to \infty$ for each $x \in [a, \omega]$, it follows that (35) tends to zero as $k \to \infty$, and so

$$\int_a^{\omega} R_k(x) f_a(x)\, dx \to 0 \quad \text{as } k \to \infty. \tag{36}$$

We can re-express this integral by invoking Taylor's Theorem [32], which guarantees the existence of a $\xi \in (a, \omega)$ such that

$$R_k(x) = \frac{e^{(k+1)}(\xi)}{(k+1)!}(x-e_a)^{k+1}.$$

Thus,

$$\int_a^\omega R_k(x) f_a(x)\,dx = \frac{e^{(k+1)}(\xi)}{(k+1)!}\int_a^\omega (x-e_a)^{k+1} f_a(x)\,dx = \frac{e^{(k+1)}(\xi)}{(k+1)!} m_{k+1}(a), \tag{37}$$

using (8). Accordingly, (36) becomes:

$$\frac{e^{(k+1)}(\xi)}{(k+1)!} m_{k+1}(a) \to 0 \quad \text{as } k \to \infty. \tag{38}$$

Finally, substituting (37) into (33) yields

$$e^\dagger(a) = e(e_a) + \frac{e''(e_a)}{2!}\sigma^2(a) + \sum_{n=3}^{k} \frac{e^{(n)}(e_a)}{n!} m_n(a) + \frac{e^{(k+1)}(\xi)}{(k+1)!} m_{k+1}(a), \tag{39}$$

which reproduces (13). The last claim in Theorem 1 then follows from (38). The proof of (14) follows from dividing (13) by e_a. □

Proof of Corollary 1.1. The assumptions of Corollary 1.1 are the same as those of Theorem 1, and equations (15)–(16) are simply the $a = 0$ case of (13)–(14). □

A.3 Hyperbolic Mortality Example

From (9) we have:

$$e_0 = \int_0^\omega s(x)\,dx = \int_0^\omega \left(1-\frac{x}{\omega}\right)^k dx = -\frac{\omega}{k+1}\left[\left(1-\frac{x}{\omega}\right)^{k+1}\right]_0^\omega = \frac{\omega}{k+1},$$

reproducing the first equation in (18). Next, using (5):

$$\begin{aligned} e(a) &= \left(1-\frac{a}{\omega}\right)^{-k}\int_a^\omega \left(1-\frac{x}{\omega}\right)^k dx \\ &= -\frac{\omega}{k+1}\left(1-\frac{a}{\omega}\right)^{-k}\left[\left(1-\frac{x}{\omega}\right)^{k+1}\right]_a^\omega \\ &= \frac{\omega}{k+1}\left(1-\frac{a}{\omega}\right)^{-k}\left(1-\frac{a}{\omega}\right)^{k+1} \\ &= \frac{\omega}{k+1}\left(1-\frac{a}{\omega}\right), \end{aligned}$$

reproducing the second equation in (18). Now, using (1):

$$\begin{aligned} e^\dagger &= \int_0^\omega \left[\frac{\omega}{k+1}\left(1-\frac{x}{\omega}\right)\right]\left[\frac{k}{\omega}\left(1-\frac{x}{\omega}\right)^{k-1}\right] dx \\ &= \frac{k}{k+1}\int_0^\omega \left(1-\frac{x}{\omega}\right)^k dx \\ &= \frac{e_0 k}{k+1} \\ &= \frac{\omega k}{(k+1)^2}, \end{aligned}$$

reproducing the third equation in (18). Finally, since $H = e^\dagger/e_0$ (recall (1)), then:

$$H = \left(\frac{k\omega}{(k+1)^2}\right)\left(\frac{k+1}{\omega}\right) = \frac{k}{k+1},$$

reproducing the last equation in (18).

B Supplementary Information

B.1 Data Analysis Methods

Because population data typically comes in discrete form, we used standard techniques to estimate $e(a)$ and $e^{\dagger}$ at time t:

$$\begin{aligned} e(a,t) &= \frac{1}{l(a,t)} \sum_{x=a}^{\omega} L(x,t), \\ e^{\dagger}(a,t) &= \frac{1}{l(a,t)} \sum_{y=a}^{\omega-1} d(y,t) \left[\frac{e(y,t)+e(y+1,t)}{2} \right]. \end{aligned}$$

where $l(x,t)$, $L(x,t)$, $d(x,t)$, and $e(x,t)$ correspond to the following life table values at age x, time t: radix at age 0, person-years lived, deaths, and remaining life expectancy. We approximated $m_n(a,t)$ at time t as:

$$m_n(a,t) = \frac{\sum_{x=a}^{\omega} (x-e_a)^n d(x,t)}{\sum_{x=a}^{\omega} d(x,t)}.$$

B.2 Data

We used data from the Human Mortality Database [23]. Specifically, we used 7,340 1-year life tables of males and females from the HMD (41 countries; 1751–2020). Table 9 shows the country-years of data included in the analysis.

B.3 Tables

Table 3. Regression coefficients for life span inequality $e^\dagger$, based on (20)–(21), for French males, National Population (1816–2018) in the Human Mortality Database [23], along with adjusted R^2-values and standard errors (S.E.). All coefficient p-values were less than 1×10^{-6}.

Parameter	$e^\dagger(0)$	$e^\dagger(0)$	$e^\dagger(0)$	$e^\dagger(40)$	$e^\dagger(40)$	$e^\dagger(40)$	$e^\dagger(80)$	$e^\dagger(80)$	$e^\dagger(80)$
Intercept	6.48551	8.04312	7.89425	4.75310	5.41384	5.89388	2.19435	2.07104	1.77078
	(0.176851)	(0.113986)	(0.10827)	(0.456569)	(0.043428)	(0.051631)	(0.018437)	(0.008537)	(0.01219)
σ^2	0.02005	0.01930	0.01632	0.03741	0.03765	0.03208	0.10401	0.10168	0.13672
	(0.00024)	(0.000129)	(0.000515)	(0.002914)	(0.000276)	(0.000506)	(0.001101)	(0.000459)	(0.001359)
m_3		0.00014	0.00018		0.00077	0.00096		0.00435	0.00744
		(0.000006)	(0.000009)		(0.000005)	(0.000016)		(0.000138)	(0.000136)
m_4			0.000002			0.00001			-0.0002
			(0.0000004)			(0.0000008)			(0.000016)
Adjusted R^2	0.9719	0.9924	0.9935	0.4478	0.9951	0.9971	0.9779	0.9963	0.9992
Regression S.E.	0.9734	0.5074	0.4689	0.2869	0.0271	0.0207	0.0743	0.0306	0.0146

Source: Authors' calculations using data from the [23].

Table 4. Regression coefficients for life table entropy H, based on (22)–(23), for French males, National Population (1816–2018) in the Human Mortality Database [23], along with adjusted R^2-values and standard errors (S.E.). All coefficient p-values were less than 1×10^{-6}.

Parameter	$H(0)$	$H(0)$	$H(0)$	$H(40)$	$H(40)$	$H(40)$	$H(80)$	$H(80)$	$H(80)$
Intercept	0.06722	0.11842	0.10236	-0.00868	0.07201	0.06140	0.02691	0.02533	0.02166
	(0.006009)	(0.004587)	(0.004181)	(0.004264)	(0.001453)	(0.001005)	(0.000251)	(0.000103)	(0.000147)
v_d	0.02450	0.02349	0.01302	0.07171	0.04487	0.05465	0.09785	0.09502	0.13105
	(0.000346)	(0.000218)	(0.001123)	(0.001882)	(0.000544)	(0.00059)	(0.001291)	(0.000483)	(0.001377)
$\widehat{m}_3$		0.00027	0.00036		0.00156	0.00102		0.00485	0.00791
		(0.000014)	(0.000016)		(0.000023)	(0.000031)		(0.000136)	(0.000132)
$\widehat{m}_4$			0.000008			-0.000021			-0.000206
			(0.0000009)			(0.0000011)			(0.000016)
Adjusted R^2	0.9612	0.9856	0.9900	0.8778	0.9950	0.9983	0.9660	0.9954	0.9990
Regression S.E.	0.0422	0.0257	0.0214	0.0047	0.0010	0.0006	0.0010	0.0004	0.0002

Source: Authors' calculations using data from the [23].

Table 5. Regression coefficients for $e^\dagger$, equations (20) and (21), for females in the Human Mortality Database [23] (41 countries, 1751–2020) along with adjusted R^2-values and standard errors (S.E.). All non-starred coefficient p-values were less than 1×10^{-6}.

Parameter	$e^\dagger(0)$	$e^\dagger(0)$	$e^\dagger(0)$	$e^\dagger(40)$	$e^\dagger(40)$	$e^\dagger(40)$	$e^\dagger(80)$	$e^\dagger(80)$	$e^\dagger(80)$
Intercept	5.77289	6.56481	6.56315	2.91733	4.73917	4.74137	2.48050	2.33112	1.98038
	(0.020873)	(0.012712)	(0.01238)	(0.0312)	(0.011863)	(0.015336)	(0.005004)	(0.003085)	(0.003748)
σ^2	0.01953	0.02026	0.01921	0.04806	0.04260	0.04256	0.08965	0.09114	0.12329
	(0.000039)	(0.00002)	(0.000077)	(0.000224)	(0.000065)	(0.000174)	(0.000219)	(0.000118)	(0.000318)
m_3		0.00010	0.00013		0.00083	0.00083		0.00321	0.00500
		(0.000001)	(0.000002)		(0.000004)	(0.00001)		(0.000033)	(0.000024)
m_4			0.000001			0.00000			-0.0002
			(0.0000001)			(0.0000004)			(0.000003)
Adjusted R^2	0.9859	0.9966	0.9967	0.9259	0.9946	0.9946	0.9786	0.9939	0.9984
Regression S.E.	0.6972	0.3445	0.3355	0.2345	0.0630	0.0630	0.0767	0.0408	0.0207

Source: Authors' calculations using data from the [23].

Table 6. Regression coefficients for $e^\dagger$, equations (20) and (21), for males in the Human Mortality Database [23] (41 countries, 1751–2020) along with adjusted R^2-values and standard errors (S.E.). All coefficient p-values were less than 1×10^{-6}.

Parameter	$e^\dagger(0)$	$e^\dagger(0)$	$e^\dagger(0)$	$e^\dagger(40)$	$e^\dagger(40)$	$e^\dagger(40)$	$e^\dagger(80)$	$e^\dagger(80)$	$e^\dagger(80)$
Intercept	6.59818	7.61094	7.58915	3.73610	5.17071	5.24487	2.23659	2.17966	1.78050
	(0.029325)	(0.019369)	(0.02015)	(0.044048)	(0.008992)	(0.013726)	(0.004142)	(0.002641)	(0.003381)
σ^2	0.01925	0.01972	0.02017	0.04400	0.03940	0.03831	0.10249	0.09798	0.13886
	(0.000053)	(0.000029)	(0.000121)	(0.000297)	(0.000055)	(0.000164)	(0.000215)	(0.000144)	(0.000332)
m_3		0.00013	0.00012		0.00079	0.00083		0.00357	0.00728
		(0.000001)	(0.000003)		(0.000002)	(0.000006)		(0.000046)	(0.000036)
m_4			0.000000			0.00000			-0.0002
			(0.0000001)			(0.0000003)			(0.000004)
Adjusted R2	0.9729	0.9919	0.9920	0.8571	0.9954	0.9955	0.9842	0.9941	0.9989
Regression S.E.	0.8688	0.4744	0.4735	0.2579	0.0462	0.0459	0.0611	0.0374	0.0163

Source: Authors' calculations using data from the [23].

Table 7. Regression coefficients for H, equations (22)–(23), for females in the Human Mortality Database [23] (41 countries, 1751–2020) along with adjusted R^2-values and standard errors (S.E.). All non-starred coefficient p-values were less than 1×10^{-6}.

Parameter	$H(0)$	$H(0)$	$H(0)$	$H(40)$	$H(40)$	$H(40)$	$H(80)$	$H(80)$	$H(80)$
Intercept	0.05916	0.07899	0.07737	0.02138	0.05718	0.05133	0.03043	0.02834	0.02426
	(0.000483)	(0.000319)	(0.000304)	(0.000383)	(0.00026)	(0.000209)	(0.000071)	(0.000035)	(0.000044)
v_d	0.02331	0.02408	0.02130	0.05744	0.05129	0.06041	0.08232	0.08411	0.11593
	(0.000044)	(0.000024)	(0.000117)	(0.000206)	(0.000082)	(0.000163)	(0.000271)	(0.00012)	(0.000321)
$\widehat{m_3}$		0.00016	0.00022		0.00146	0.00081		0.00390	0.00555
		(0.000002)	(0.000003)		(0.000009)	(0.000013)		(0.000032)	(0.000023)
$\widehat{m_4}$			0.000002			-0.000026			-0.000206
			(0.0000001)			(0.0000004)			(0.000003)
Adjusted R^2	0.9872	0.9966	0.9970	0.9548	0.9944	0.9972	0.9619	0.9926	0.9981
Regression S.E.	0.0200	0.0103	0.0096	0.0042	0.0015	0.0011	0.0010	0.0004	0.0002

Source: Authors' calculations using data from the [23].

Table 8. Regression coefficients for H, equations (22)–(23), for males in the Human Mortality Database [23] (41 countries, 1751–2020) along with adjusted R^2-values and standard errors (S.E.). All coefficient p-values were less than 1×10^{-6}.

Parameter	$H(0)$	$H(0)$	$H(0)$	$H(40)$	$H(40)$	$H(40)$	$H(80)$	$H(80)$	$H(80)$
Intercept	0.07028	0.09932	0.09930	0.02510	0.06669	0.05615	0.02732	0.02658	0.02181
	(0.00072)	(0.000537)	(0.000525)	(0.000628)	(0.000305)	(0.000236)	(0.000056)	(0.000031)	(0.00004)
v_d	0.02377	0.02409	0.02178	0.05711	0.04622	0.05904	0.09664	0.09118	0.13258
	(0.000062)	(0.000036)	(0.000187)	(0.000302)	(0.000113)	(0.000183)	(0.000249)	(0.000148)	(0.000331)
$\widehat{m_3}$		0.00020	0.00024		0.00146	0.00082		0.00417	0.00775
		(0.000002)	(0.000004)		(0.000008)	(0.00001)		(0.000045)	(0.000034)
$\widehat{m_4}$			0.000002			-0.000029			-0.000206
			(0.0000001)			(0.0000004)			(0.000004)
Adjusted R^2	0.9760	0.9920	0.9923	0.9072	0.9906	0.9963	0.9762	0.9929	0.9987
Regression S.E.	0.0277	0.0160	0.0156	0.0051	0.0016	0.0010	0.0008	0.0004	0.0002

Source: Authors' calculations using data from the [23].

Table 9. Life tables from the Human Mortality Database [23] by country and years. Totals are shown for female life tables; totals for male life tables are the same.

	Accronym	Country	Start	End	Total life tables
1	AUS	Australia	1921	2018	98
2	AUT	Austria	1947	2019	73
3	BEL	Belgium	1841	2018	178
4	BGR	Bulgaria	1947	2017	71
5	BLR	Belarus	1959	2018	60
6	CAN	Canada	1921	2018	98
7	CHE	Switzerland	1876	2018	143
8	CHL	Chile	1992	2017	26
9	CZE	Czech Republic	1950	2019	70
10	DEUTE	Germany, Former Democratic Republic	1956	2017	62
11	DEUTW	Germany, Former Federal Republic	1956	2017	62
12	DNK	Denmark	1835	2020	186
13	ESP	Spain	1908	2018	111
14	EST	Estonia	1959	2019	61
15	FIN	Finland	1878	2020	143
16	FRATNP	France	1816	2018	203
17	GBRTENW	United Kingdom, England and Wales	1841	2018	178
18	GRC	Greece	1981	2017	37
19	HKG	Hong Kong	1986	2019	34
20	HRV	Croatia	2001	2019	19
21	HUN	Hungary	1950	2017	68
22	IRL	Ireland	1950	2017	68
23	ISR	Israel	1983	2016	34
24	ITA	Italy	1872	2018	147
25	JPN	Japan	1947	2019	73
26	KOR	Republic of Korea	2003	2018	16
27	LTU	Lithuania	1959	2019	61
28	LUX	Luxembourg	1960	2019	60
29	LVA	Latvia	1959	2019	61
30	NLD	Netherlands	1850	2019	170
31	NOR	Norway	1846	2020	175
32	NZL_NP	New Zealand	1948	2013	66
33	POL	Poland	1958	2019	62
34	PRT	Portugal	1940	2020	81
35	RUS	Russian Federation	1959	2014	56
36	SVK	Slovakia	1950	2017	68
37	SVN	Slovenia	1983	2017	35
38	SWE	Sweden	1751	2019	269
39	TWN	China: Province of Taiwan only	1970	2019	50
40	UKR	Ukraine	1959	2013	55
41	USA	United States of America	1933	2019	87

B.4 Additional Plots

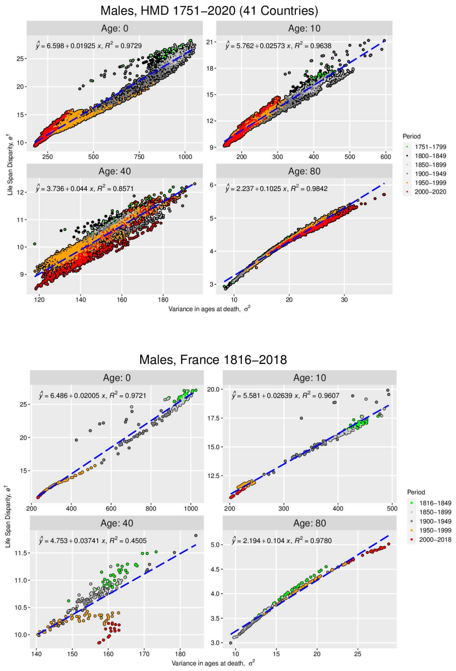

Fig 3. Life Span Disparity versus Variance in Age at Death for Males in the Human Mortality Database and French National Population. Plots of the a-truncated life span disparity, $e^{\dagger}(a)$, versus the a-truncated variance in age at death, $\sigma^2(a)$ for (a) males in 41 countries in the Human Mortality database [23] (3,670 1-year life tables; 1751–2020) and (b) males in France, National Population in the Human Mortality database [23] (203 1-year life tables; 1816–2018). The dashed lines are the plots of the best-fit regression lines; the error statistics associated with panel (a) are detailed in Table 6, and those associated with panel (b) are detailed in Table 3.

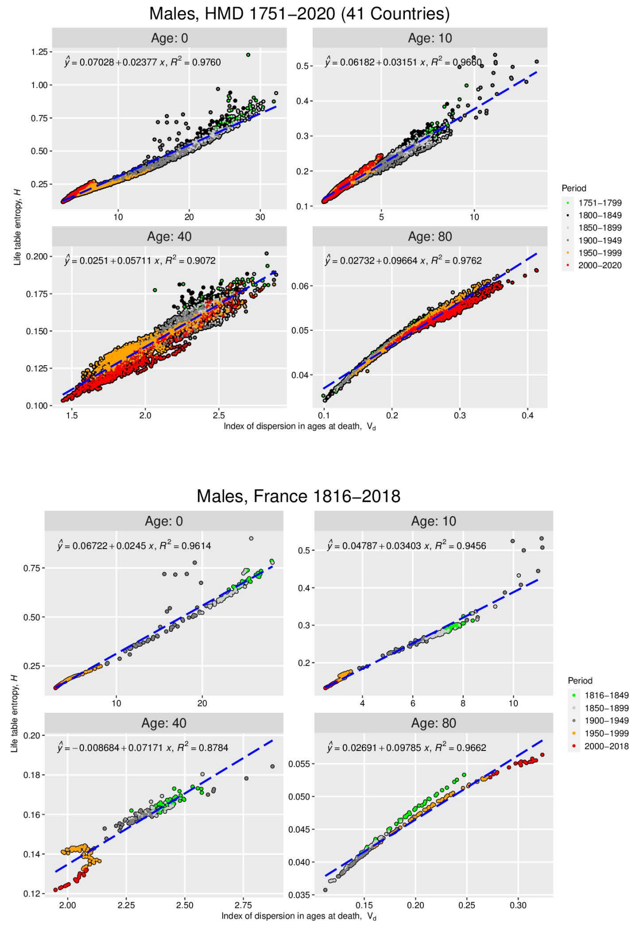

Fig 4. Life Table Entropy versus Index of Dispersion of Deaths Distribution for Males in the Human Mortality Database and French National Population. Plots of the a-truncated life table entropy, $H(a)$, versus the a-truncated index of dispersion of age at death, $v_d(a)$ for (a) males in 41 countries in the Human Mortality database [23] (3,670 1-year life tables; 1751–2020) and (b) males in France, National Population in the Human Mortality database [23] (203 1-year life tables; 1816–2018). The dashed lines are the plots of the best-fit regression lines; the error statistics associated with panel (a) are detailed in Table 8, and those associated with panel (b) are detailed in Table 4.

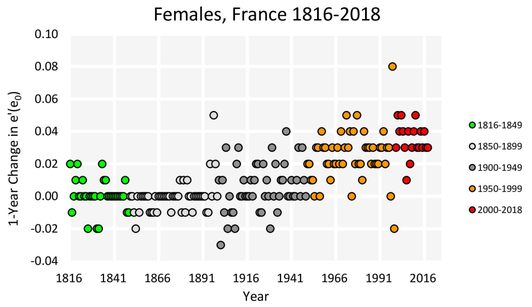

Fig 5. One-year change in $e'(e_0)$—the discrete approximation of $e''(e_0)$—for French females in the Human Mortality database [23] (203 1-year life tables for males and females; 1816–2018) since 1816.

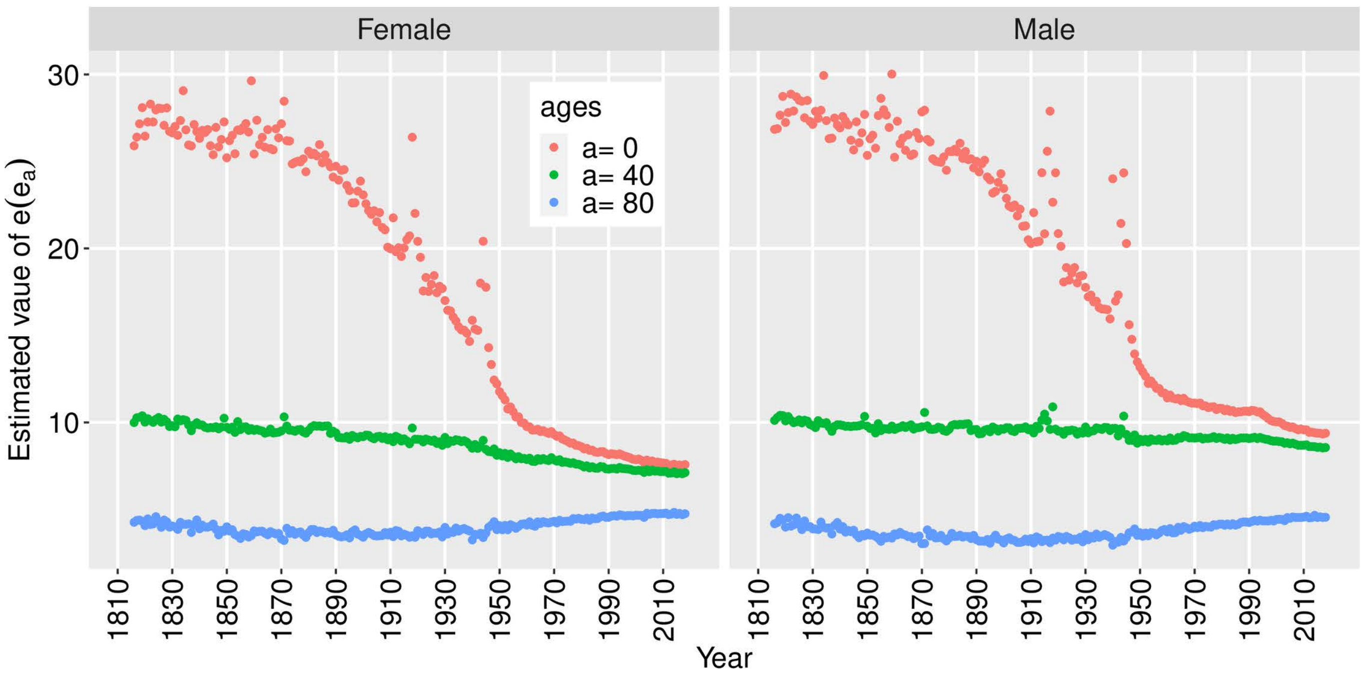

Fig 6. Mean life span of individuals in the French National Population that have already survived to age a, e_a ($a = 0$, 10, 80) in the Human Mortality database [23]. Note: Values are estimated by fitting a third-degree polynomial to the life expectancy column in a life table separately for each year, and using that polynomial to predict the value at e_a for each a-value.

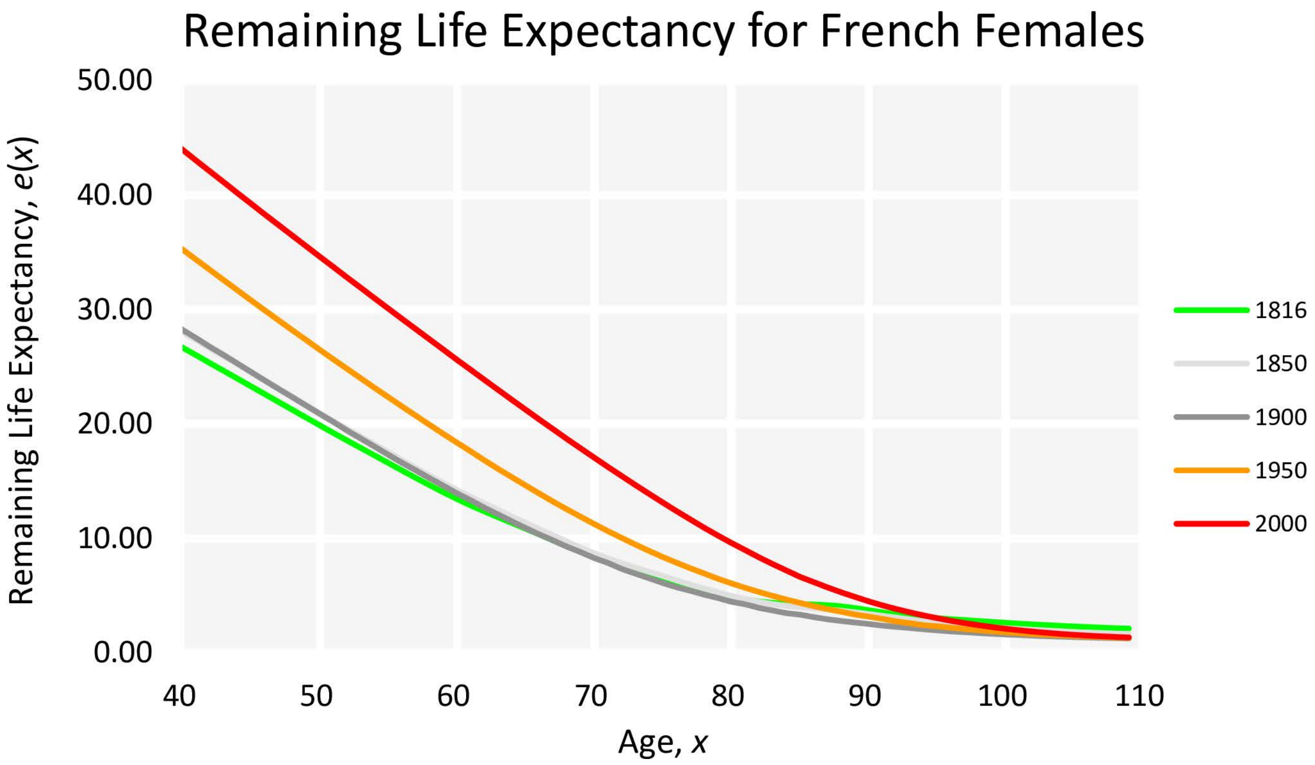

Fig 7. Remaining life expectancy function $e(x)$ for $x \geq 40$ for French females, National Population, in the Human Mortality database [23] for years 1816, 1850, 1900, 1950, and 2000.

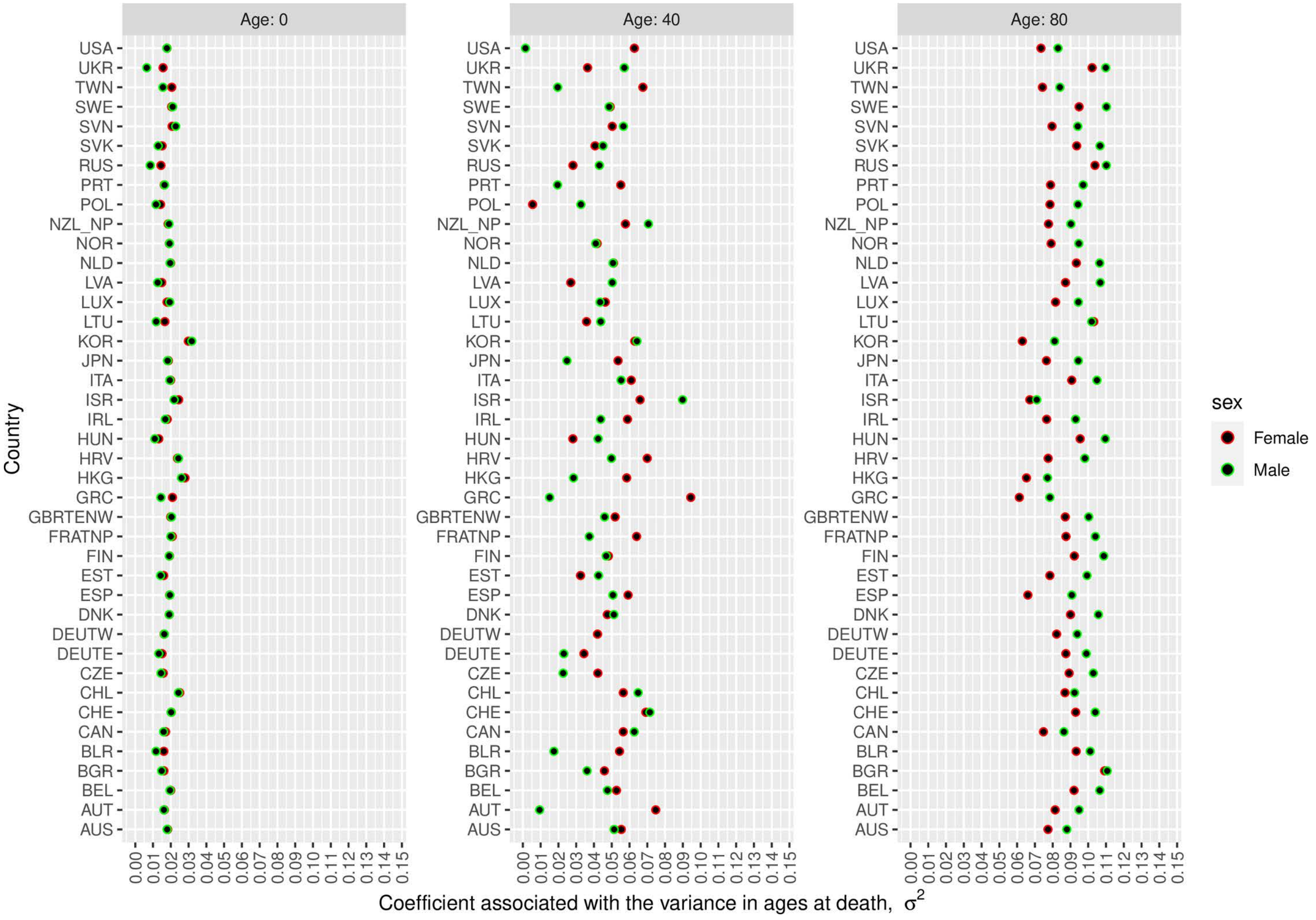

Fig 8. Coefficient estimates for σ^2 in regressions for $e^\dagger$ from separate models by country-sex-age in the Human Mortality database [23]. The values shown are from the second-order truncation: $e^\dagger(t) = \alpha + \beta \cdot \sigma^2(t) + \epsilon(t)$, see equation (24).

Note: See Table 9 for country names.
